# A comparative study of efficiency droop and internal electric field for InGaN blue lighting-emitting diodes on silicon and sapphire substrates

**DOI:** 10.1038/srep44814

**Published:** 2017-04-12

**Authors:** H. Y. Ryu, K. S. Jeon, M. G. Kang, H. K. Yuh, Y. H. Choi, J. S. Lee

**Affiliations:** 1 Department of Physics, Inha University, Incheon 22212, Korea; 2 LG Electronics Advanced Research Institute, Seoul 06763, Korea

## Abstract

We investigated the efficiency droop and polarization-induced internal electric field of InGaN blue light-emitting diodes (LEDs) grown on silicon(111) and *c*-plane sapphire substrates. The efficiency droop of the LED sample grown on silicon substrates was considerably lower than that of the identically fabricated LED sample grown on sapphire substrates. Consequently, the LED on silicon showed higher efficiency at a sufficiently high injection current despite the lower peak efficiency caused by the poorer crystal quality. The reduced efficiency droop for the LED on silicon was attributed to its lower internal electric field, which was confirmed by reverse-bias electro-reflectance measurements and numerical simulations. The internal electric field of the multiple quantum wells (MQWs) on silicon was found to be reduced by more than 40% compared to that of the MQWs on sapphire, which resulted in a more homogenous carrier distribution in InGaN MQWs, lower Auger recombination rates, and consequently reduced efficiency droop for the LEDs grown on the silicon substrates. Owing to its greatly reduced efficiency droop, the InGaN blue LED on silicon substrates is expected to be a good cost effective solution for future lighting technology.

Since the first demonstration of GaN-based blue light-emitting diodes (LEDs) in the early 1990s, there have been remarkable achievements in GaN-based LEDs over the last two decades^1^. On the other hand, despite such technological achievements and successful commercialization, some scientific issues limiting the efficiency of LEDs still remain^2^,^3^. For example, the emission efficiency of blue LEDs with InGaN multiple-quantum-well (MQW) active layers decreases significantly with increasing injection current. This “efficiency droop” phenomenon results in limited efficiency at high current densities^4^,^5^,^6^. In addition to the efficiency issue, the high cost of the current LED light sources is still a factor hindering their penetration into the general lighting market.

Although the true origin of the efficiency droop has not yet been clearly identified, Auger recombination^7^,^8^, electron leakage^9^,^10^, and carrier delocalization^11^,^12^ have been proposed as the mechanism of the efficiency droop in InGaN LEDs. In addition, the polarization-induced internal electric field in InGaN MQWs is known to have a strong influence on the efficiency droop^4^,^5^,^6^. As the strength of the internal electric field increases, electron leakage from the MQWs to the p-GaN layer increases and hole injection into the MQW active region becomes increasingly inefficient, which aggravates the efficiency droop problem. In addition, the internal electric field was reported to increase the Auger recombination rate, which also leads to an increase in efficiency droop^13^,^14^,^15^. Therefore, a number of studies have been devoted to reducing the polarization-induced field in InGaN QWs.

Recently, it was reported that the strain and internal polarization of InGaN MQWs grown on silicon substrates can be reduced greatly based on electro-reflectance (ER) measurements, micro-Raman spectroscopy, and high-resolution transmission electron microscopy (HR TEM)^16^,^17^. This suggests that the efficiency droop can be lower in InGaN LEDs grown on silicon substrates. Moreover, the GaN-on-Si technology is expected to have great potential to unlock the cost savings for the high volume manufacturing of LEDs. Silicon has been considered as an attractive substrate material for LEDs because of its inexpensive large-sized wafers. Several groups have reported high-performance GaN-based LEDs grown on silicon substrates overcoming the large mismatch in the lattice constant and thermal expansion coefficients between GaN and silicon^18^,^19^,^20^,^21^, and the successful commercialization of GaN LEDs on silicon substrates is expected to be realized in the near future. However, the efficiency droop of InGaN LEDs on silicon substrates have been relatively unexplored compared to the extensive results on the efficiency droop of InGaN LEDs on sapphire substrates.

In this paper, we compare the efficiency droop and internal electric field of InGaN blue LEDs grown on silicon and sapphire substrates. LED structures with identical MQW active layers were grown on silicon and sapphire substrates, and LED chips with a vertical injection type were fabricated. Then, the electro-luminescence (EL) efficiency and efficiency droop of the LEDs on silicon and sapphire were compared. Reverse-biased ER spectroscopy was used to compare the polarization-induced internal electric field and to understand the difference in the efficiency droop. In addition, numerical simulations were performed to interpret the experimental results on the internal electric fields and efficiency droop. To our knowledge, this is the first systematic study comparing the efficiency droop of InGaN LEDs grown on sapphire and silicon substrates in the view point of the internal electric fields.

## Results

**Figure 1:**
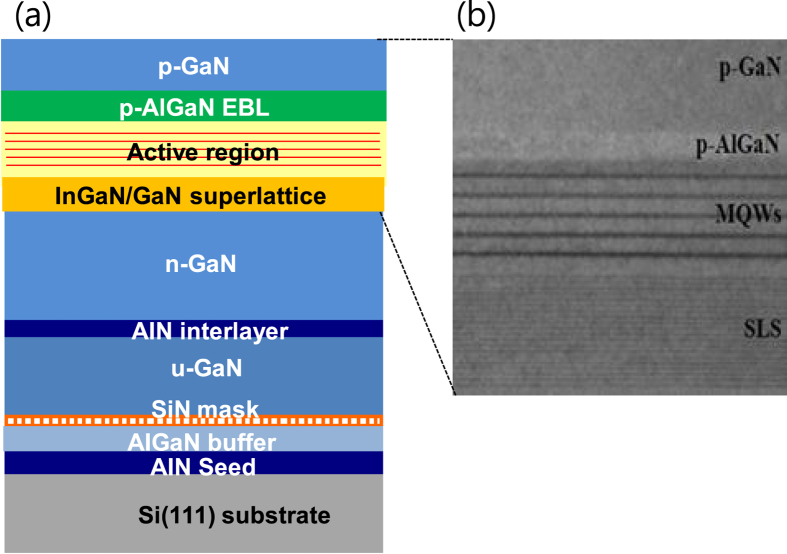
Schematic diagram and TEM image of LED layer structures grown on a silicon(111) substrate. (**a**) Schematic cross-section diagram of the epitaxial layer structures grown on a silicon(111) substrate. Several layers were introduced between the silicon substrate and n-GaN layer for stress control. (**b**) Cross-sectional TEM image of the epitaxial layers around the MQW region for the wafer grown on the silicon substrate.

The epitaxial layers were grown on 6-inch silicon(111) and sapphire substrates using metal organic chemical vapor deposition. For growth on silicon substrates, the control of stress is important to reduce the number of threading dislocations due to the large mismatch in the lattice constant and the thermal expansion coefficients between GaN and silicon. Therefore, several layers were introduced between the silicon substrate and n-GaN layer for stress control. Figure 1(a) presents a schematic diagram of the epitaxial layer structures grown on a silicon substrate. First, a 180-nm AIN seed layer and an 800-nm AlGaN step-graded buffer layer were grown on the silicon substrate. The SiN masking layer was then deposited, and a 1-μm GaN coalescence layer, a 15-nm AIN interlayer, and a 1-μm unintentionally-doped GaN layer were then grown. Compared to the template layers on the silicon substrate, the GaN template grown on the *c*-plane sapphire substrate consisted of a 30-nm low-temperature buffer layer and a 2-μm GaN layer.

**Figure 2:**
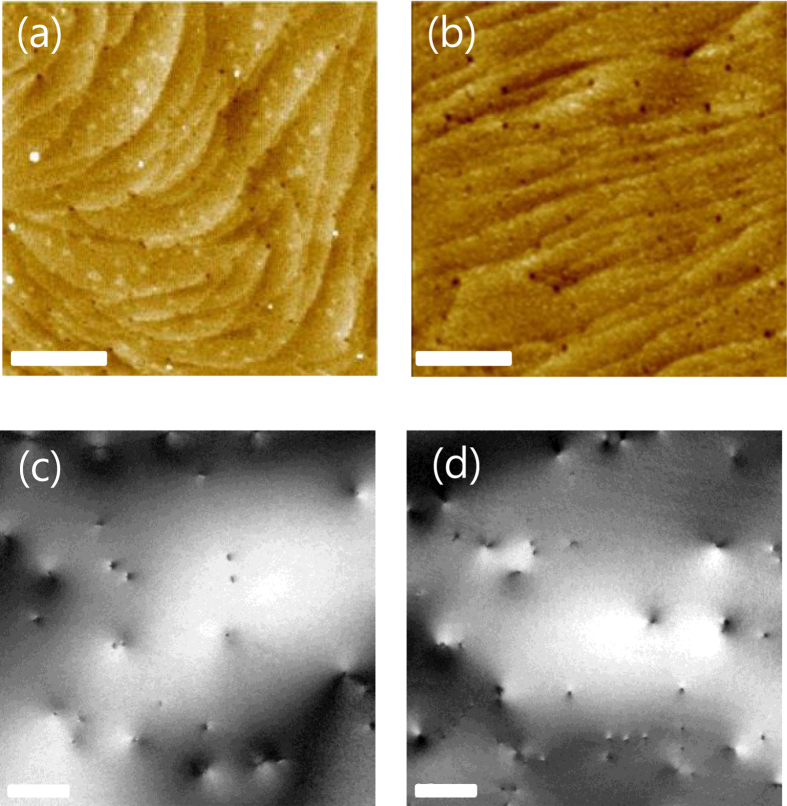
AFM and plan-view TEM images of GaN templates on sapphire and silicon substrates. AFM images of a GaN template on (**a**) sapphire (TDD: 3.8 × 10^8^ cm^−2^) and (**b**) silicon (TDD: 5.3 × 10^8^ cm^−2^). Plan-view TEM images of a GaN template on (**a**) sapphire and (**b**) silicon. The scale bar of both AFM and plan-view TEM images is 0.5 μm.

The crystal quality of the GaN layer was evaluated from the x-ray (102) omega rocking curves obtained by x-ray diffraction (XRD) the threading dislocation density (TDD) determined by atomic force microscopy (AFM) and transmission electron microscopy (TEM). The full width at half maximum value was measured to be 320 and 350 arcsec for the GaN on sapphire and the GaN on silicon, respectively. Figure 2 shows the AFM and the plan-view TEM images of GaN templates grown on sapphire and silicon substrates. The TDD determined by the AFM measurements was estimated to be 3.8 × 10^8^ cm^−2^ for the GaN on sapphire and 5.3 × 10^8^ cm^−2^ for the GaN on silicon. The TDDs determined by using the plane-view TEM was also similar to those determined by using the AFM. The XRD and TDD measurements suggest that the crystal quality of the GaN on sapphire is slightly better than that of the GaN on silicon.

LED layer structures were grown on each template on the silicon and sapphire substrates. The LED epitaxial layer structures consisted of a 2.5-μm n-GaN layer, strain relief layers with a short-period In_0.05_Ga_0.95_N/GaN superlattice, MQW active region, a 15-nm Al_0.15_Ga_0.85_N electron-blocking layer, and a 90-nm p-GaN layer. The MQW layers consisted of five periods of a 2.5-nm In_0.15_Ga_0.85_N well separated by an 8-nm GaN barrier layer. Figure 1(b) shows a cross-sectional transmission electron microscope image of the epitaxial layers around the MQW region for the LED on silicon. No propagation of threading dislocations through the LED layer structures was observed.

The LED chip structures were then fabricated in the type of vertical current injection with chip dimensions of 1 × 1 mm^2^. A chemical lift-off and a laser lift-off process were employed to remove the silicon and sapphire substrates, respectively. The exposed n-GaN surface was roughened by a KOH solution for efficient light extraction. The fabricated LED chips were packaged as a type of surface-mount device and the optical characteristics were measured. The peak wavelength was observed at around 445 nm for both LED samples with silicon and sapphire substrates.

**Figure 3:**
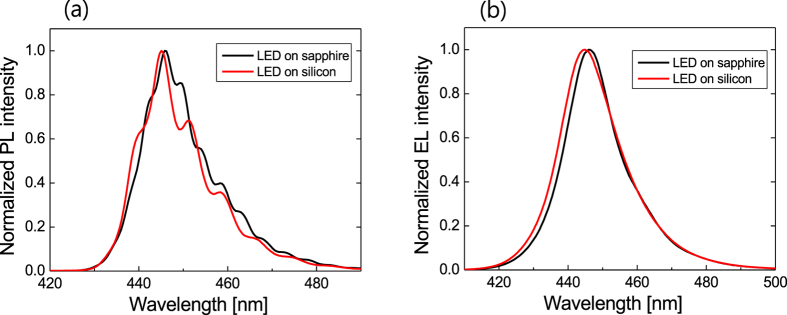
Comparison of PL and EL spectra for the LED on silicon and sapphire substrates. (**a**) Photoluminescence (PL) and (**b**) electroluminescence (EL) spectra of the LED on sapphire (black line) and the LED on silicon (red line). The peak wavelength appear around 445 nm for both LEDs on sapphire and silicon.

Figure 3 compares the photoluminescence (PL) and the electroluminescence (EL) spectra for LED structures on sapphire and silicon. The intensity of each spectrum was normalized to its peak value. The modulation of the PL spectrum in Fig. 3(a) was caused by the formation of a Fabry-Perot resonator created by GaN/sapphire (or GaN/silicon) and GaN/air interfaces since the PL spectra of Fig. 3(a) were measured on a wafer before chip fabrication. In the EL spectra in Fig. 3(b) measured at 100 mA after the chip fabrication, the spectral modulation disappeared due to the textured surface. The peak wavelength of both LEDs on sapphire and silicon was observed around 445 nm, implying that the thickness and the In composition for MQWs on sapphire and silicon are almost identical to each other. The EL spectrum of the LED on silicon is only slightly broader than that of the LED on sapphire. This suggests that the MQW quality of the LED on sapphire should be a little better than that of the LED on silicon, which was also expected from the XRD and TDD measurements.

**Figure 4:**
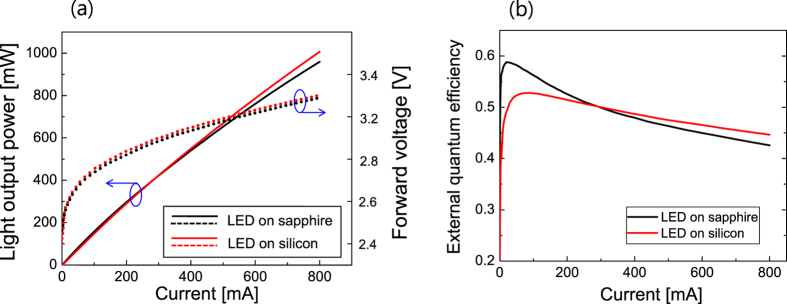
Comparison of the light output power, forward voltage and external quantum efficiency for the LED on silicon and sapphire substrates. (**a**) Light output power (solid line) and forward voltage (dotted line) of the LED on sapphire (black line) and the LED on silicon (red line) as a function of the injection current. (**b**) External quantum efficiency of the LED on sapphire (black line) and the LED on silicon (red line) as a function of the injection current. The LED on silicon exhibits lower peak EQE but reduced efficiency droop.

Figure 4 presents light output power (LOP), injection current versus forward voltage (I-V curve), and external quantum efficiency (EQE) of the measured LED samples as a function of the injection current up to 800 mA. The I-V curves for the LEDs on sapphire and silicon are shown as dotted lines in Fig. 4(a). The difference in the forward voltage for the LEDs on sapphire and silicon was only ~0.1 V up to 800 mA, implying that the electrical characteristics of the LEDs on sapphire and silicon are almost identical. When the current was <300 mA, the LOP of both LED samples grown on silicon and sapphire were similar. On the other hand, the EQE curves show that the EQE of the LED on sapphire was higher than that of the LED on silicon when the current was <300 mA. The peak EQE for the LED on silicon and the LED on sapphire was 0.588 and 0.528, respectively. This means that, for a relatively low injection current, the LED on sapphire is more efficient than the LED on silicon because of the better crystal quality of the LED on sapphire, as revealed by the XRD and TDD measurements. That is, the Shockley-Read-Hall (SRH) recombination rate of the LED on sapphire is believed to be lower than that of the LED on silicon.

For injection currents larger than 300 mA, however, the LOP and EQE of the LED on silicon was higher than those of the LED on sapphire. When the injection current was 800 mA, the LOP of the LED on silicon and sapphire was 1007 mW and 960 mW, respectively. The EQE curves show that the efficiency droop of the LED on silicon is reduced substantially compared to that of the LED on sapphire. The droop ratio, which is defined as the difference between the peak EQE and the EQE at 800 mA normalized to the peak EQE^22^, was obtained to be 38% and 18%, respectively. The higher SRH recombination rate for the LED on silicon can partly explain the reduced efficiency droop^23^. However, it cannot explain the higher EQE for the LED on silicon when the current is larger than 300 mA. Another reason for the reduced droop is attributed to the lower strength in the strain and internal electric field of the LED on silicon, as mentioned before. Therefore, the internal electric fields of the LED on silicon and sapphire were compared experimentally to understand the difference in the efficiency droop.

**Figure 5:**
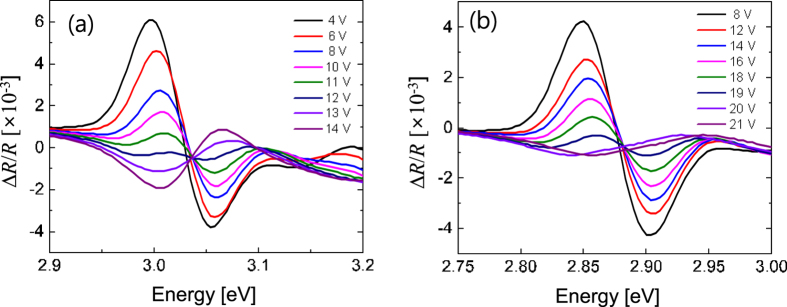
Comparison of electro-reflectance (ER) spectra for the LED on silicon and sapphire. ER spectra of (**a**) the LED on silicon and (**b**) the LED on sapphire with increasing reverse bias voltage. The flat-band voltage, where the phase angle of the ER spectra is changed by 180°, was determined to be −12 V for the LED on silicon and −20 V for the LED on sapphire.

It has been reported that the strain of the GaN template on silicon substrates is lower than that of the GaN template on sapphire substrates based on the micro-Raman spectroscopy and the HR TEM measurement. The strain characteristics and the internal electric field of InGaN MQWs can be analyzed by using the reverse-bias ER spectroscopy^17^,^24^,^25^,^26^,^27^,^28^,^29^. In the ER experiments, the relative change in reflectivity was measured while modulating the internal electric field. Figure 5 presents the ER spectra of the LED samples on silicon and sapphire with increasing reverse bias voltage. The internal electric field in the MQWs decreased with increasing reverse bias, resulting in a blue shift due to the quantum confined Stark effect. A flat-band condition is achieved when the applied reverse bias reaches a compensation voltage that can cancel the internal electric field. At this point, the phase angle of the ER spectra is changed by 180° and the peak amplitude becomes negative^26^,^27^. The flat-band voltage was determined to be −20 V for the LED on sapphire and −12 V for the LED on silicon, as shown in Fig. 5. The lower magnitude of the flat-band voltage for the LED on silicon indicates that the internal electric field of the LED on silicon is weaker than that of the LED on sapphire, which is due to the reduced efficiency droop for the LED on sapphire. The internal electric fields have been usually determined from a simple formula relating the internal electric fields with the flat-band voltage and the depletion width each of which can be measured by the ER and the capacitance-voltage measurement^17^,^25^,^26^,^27^. However, it is not easy to use the simple formula for LED structures with complicated superlattice layers^30^. Therefore, we adopt numerical simulations employing the exact LED layer structures and doping profile along with the ER measurement data to determine the internal electric field in this work.

**Figure 6:**
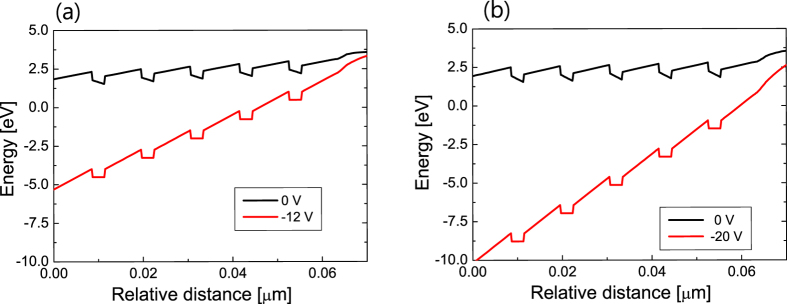
Simulated conduction band diagram around the MQW region for the LED on silicon and sapphire. Conduction band diagram of (**a**) the LED on silicon and (**b**) the LED on sapphire for the zero bias (black line) and the flat band voltage (red line). The flat-band voltage obtained from the ER measurements is −12 V for the LED on silicon and −20 V for the LED on sapphire. From the simulated conduction band diagram, the internal electric field in the InGaN QWs under zero bias was obtained to be −0.92 MV/cm for the LED on silicon and −1.50 MV/cm for the LED on sapphire.

To interpret the experimentally measured ER spectra and EQE curves, a semiconductor device simulation software, APSYS was employed^31^. The magnitude of the internal electric field can be determined by simulation with the measured flat-band voltage. For a given flat-band voltage, −12 V for the LED on silicon and −20 V for the LED on sapphire, the energy band and electric field in MQW region were calculated by varying the interface charge density. Then, the interface polarization charge density for achieving the flat-band condition can be determined. Figure 6 shows the simulated conduction band diagram around the MQW region for the LED on silicon and the LED on sapphire. The energy band of QWs is almost flat when the reverse bias voltage corresponds to the flat band voltage. Under zero bias, the internal electric field in the InGaN QWs grown on the silicon and the sapphire substrate was found to be −0.92 MV/cm and −1.50 MV/cm, respectively. Each of these values corresponds to 58% and 85% of the theoretically value predicted by the model reported in ref. 32. That is, the internal electric field of the QWs on silicon is reduced by ~40% compared to that of the QWs on sapphire.

**Figure 7:**
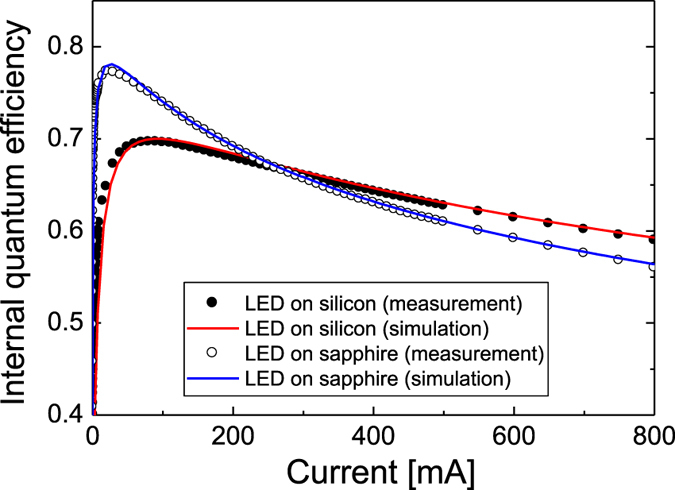
Simulated IQE curves along with the measured efficiency data for the LED on silicon and sapphire. (**a**) The simulated IQE curve (red line) and the measured efficiency data (closed black circles) for the LED on silicon. (**b**) The simulated IQE curve (blue line) and the measured efficiency data (open circles) for the LED on sapphire. Best fit was obtained when the SRH carrier lifetime (*τ*_SRH_) is 45 ns and the Auger recombination coefficient (*C*) is 8 × 10^31 ^cm^6^/s for the LED on silicon and *τ*_SRH_ is 105 ns and *C* is 7 × 10^31^ cm^6^/s for the LED on sapphire.

With the resulting polarization charge density, the internal quantum efficiency (IQE) of the LED structures was simulated as a function of the injection current. Since the SRH and Auger recombination rates have a strong influence on the peak efficiency and efficiency droop of the InGaN LEDs, the SRH carrier lifetime (*τ*_SRH_) and the Auger recombination coefficient (*C*) were used as parameters to fit the measured efficiency curves. The simulated IQE curves were compared with the measured EQE curves shown in Fig. 4. Assuming that the light extraction efficiency (LEE) is constant regardless of the injection current, the shape of the EQE curve should be identical to that of the IQE curve. Figure 7 presents the simulated IQE curves along with the measured data for the LED samples on silicon and sapphire. The simulated IQE curves shows a good fit with the measured data when *τ*_SRH_ is 45 ns and *C* is 8 × 10^31^ cm^6^/s for the LED on silicon and *τ*_SRH_ is 105 ns and *C* is 7 × 10^31^ cm^6^/s for the LED on sapphire. No electron leakage from the MQWs to p-GaN was observed even at 800 mA for both LED samples. The peak IQE was 0.697 and 0.773 for the LED on silicon and on sapphire, respectively. From the measured peak EQE values shown in Fig. 4, the LEE was calculated to be ~76% for both LED samples.

Note that *τ*_SRH_ of the LED on silicon is less than half that of the LED on sapphire, while the coefficient *C* is similar for the two samples. The lower *τ*_SRH_ value for the LED on silicon is attributed to the higher density of nonradiative recombination centers caused by the poorer crystal quality of the GaN on silicon, as expected. The small difference in the coefficient *C* between the LED on silicon and sapphire implies that the internal electric field does not have much influence on the Auger recombination rate. Despite its lower *τ*_SRH_ value, the LED on silicon showed higher efficiency when the injection current was >300 mA as a result of the reduced efficiency droop.

**Figure 8:**
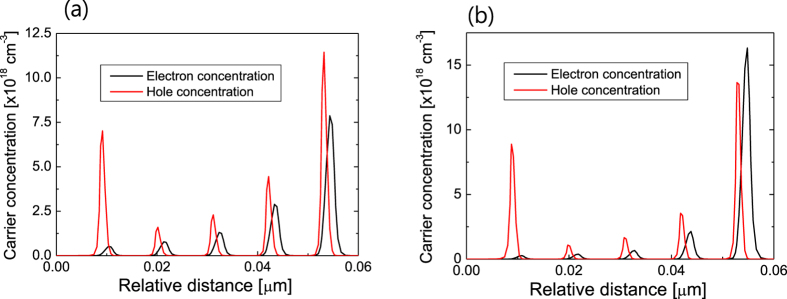
Comparison of carrier concentration distribution in MQWs for the LED on silicon and sapphire. Simulated electron concentration (black lines) and hole concentration (red lines) distribution in MQWs for (**a**) the LED on silicon and (**b**) the LED on sapphire. The electron and hole distribution of the MQWs on sapphire is more inhomogeneous than that of the MQWs on silicon.

The reduced efficiency droop for the LED on sapphire is believed to have originated from its lower internal electric field in the QWs. As the internal electric field is increased, the carrier distribution in the MQWs becomes increasingly inhomogeneous, which could increase the efficiency droop^33^,^34^. Figure 8 compares the simulated electron and hole concentration distribution in MQWs for the LED on silicon and the LED on sapphire. The electron and hole distribution of the MQWs on sapphire is more inhomogeneous than that of the MQWs on silicon due to the larger internal electric field. The inhomogeneous carrier distribution results in a large increase in the Auger recombination at the p-side QWs, which increases the efficiency droop. In addition, the internal electric field makes the electron and hole wavefunctions distributed near the edge of a QW in the opposite direction. As shown in Fig. 8, electron and hole concentration of the MQWs on sapphire is more separated compared with that of the MQWs on silicon. As the strength of the internal field increases, the spatial overlap of the electron and hole wavefunctions decreases, which effectively reduces carrier recombination region inside the QW^35^,^36^. As a result of this decrease in the effective active region, the Auger recombination rate increases with increasing internal field, leading to an increase in the efficiency droop.

The simulation results indicate that the reduced efficiency droop for the LED on silicon is a consequence of the lower internal electric field compared to the LED on sapphire. Owing to its greatly reduced efficiency droop, the InGaN blue LED grown on the silicon substrate can be used advantageously in high-power and high-efficiency illumination sources operating at high current densities.

## Conclusion

This study compared the efficiency droop and polarization-induced internal electric field of InGaN blue LEDs grown on silicon(111) and *c*-plane sapphire substrates. The LED samples with an identical active layer and chip structures were fabricated and the EL efficiency and efficiency droop characteristics were compared. The efficiency droop of the LED on silicon was significantly lower than that of the LED on sapphire, resulting in a higher EQE for the LED on silicon at an injection current >300 mA despite its lower peak EQE caused by the lower crystal quality. Reverse-bias ER measurements and simulation results of the QW internal field showed that the polarization-induced internal electric field of the LED on silicon was found to be reduced by more than 40% compared to that of the LED on sapphire, which is believed to be a main reason for the reduced efficiency droop of the LED on silicon. The numerical simulations of the IQE and the carrier distribution in MQWs also confirmed the experimental results. Owing to its greatly reduced efficiency droop and the low manufacturing cost, the InGaN blue LED grown on silicon substrates is expected to be a good cost effective solution for high-efficiency solid state lighting.

## Methods

### Device characterization

In the PL measurement, a laser diode (LD) emitting at 405 nm was used as an excitation source to enable photo-excited carrier generation only in the InGaN QW layers. The pump LD was operated under continuous-wave (CW) mode at 25 °C and the incident excitation power of the pump LD was 10 mW for the PL spectrum measurement. A spectrometer (Oceanoptics, USB4000) was used to measure the spectra. In the EL measurement, the LED sample was packaged and placed on a thermal stage. The temperature of the thermal stage was maintained at 25 °C using a thermo-electric-cooler during the measurements. The optical and electrical characteristics of the LED samples were measured using an LED characterization system with a calibrated integrated sphere. The LED sample was operated under pulsed current injection with a 0.5-ms pulse width and a 1% duty cycle to minimize the self-heating effects.

### Electroreflectance measurement

In the ER experiments, an LED sample was illuminated by a 150-W Xe arc lamp combined with a 0.25-m dual-grating monochromator for a probe beam. The probe beam was used to illuminate vertically on the sample. The internal electric field was modulated by a function generator with a modulation voltage of 100 mV at 1 KHz. The reflected beam was detected by a photomultiplier tube. The AC and DC component of reflected light were measured by a lock-in amplifier and a DC voltage meter, respectively. The relative change in reflectivity, ∆*R/R* was obtained by dividing the AC component by the DC component.

### Device simulation

The internal electric field and the IQE curves were simulated using a commercial software (Crosslight, APSYS). The APSYS program self-consistently solves the QW band structures, radiative and nonradiative carrier recombination, and the drift and diffusion equation of the carriers. In the carrier recombination model of APSYS, the radiative recombination rate is calculated by integrating the spontaneous emission spectrum with a Lorentzian line-shape function. The layer thickness and the doping concentration of the n-GaN, superlattice SRL, MQW active region, EBL, and p-GaN are identical to those of the actually grown layer structures. The conduction band offset of the InGaN/GaN and AlGaN/GaN layers was set to 0.7^37^. The Mg doping concentration of the p-GaN and p-AlGaN layers was 1 × 10^19^ cm^−3^, and the Si doping concentration of the n-GaN layer was 5 × 10^18^ cm^−3^. A background electron concentration of 1 × 10^17^ cm^−3^ was assumed in the unintentionally doped QW and barrier layers^38^. The incomplete ionization of Mg acceptors and the field ionization model were included, and the AlGaN acceptor energy was linearly scaled from 170 meV in p-GaN to 470 meV in p-AlN^39^. The internal electric field induced by spontaneous and piezo-electric polarizations at the hetero-interfaces, InGaN/GaN and AlGaN/GaN, are also included. The polarization-induced internal electric field is modeled by placing the surface charge density at the hetero-interfaces.

## Additional Information

**How to cite this article:** Ryu, H. Y. *et al*. A comparative study of efficiency droop and internal electric field for InGaN blue lighting-emitting diodes on silicon and sapphire substrates. *Sci. Rep.* **7**, 44814; doi: 10.1038/srep44814 (2017).

**Publisher's note:** Springer Nature remains neutral with regard to jurisdictional claims in published maps and institutional affiliations.
